# Investigating the Factors Affecting the Commutability of Hemoglobin A_1C_ Frozen Pooled Blood Materials

**DOI:** 10.1002/jcla.70125

**Published:** 2025-10-31

**Authors:** Kanokwan Ngueanchanthong, Napaporn Apiratmateekul, Supaporn Suparak, Nam K. Tran, Wanvisa Treebuphachatsakul

**Affiliations:** ^1^ Reference Material and Medical Laboratory Innovation Research Unit, Faculty of Allied Health Sciences Naresuan University Phitsanulok Thailand; ^2^ Department of Medical Sciences, Ministry of Public Health Nonthaburi Thailand; ^3^ Department of Medical Technology, Faculty of Allied Health Sciences Naresuan University Phitsanulok Thailand; ^4^ Department of Pathology, Laboratory Medicine Section, School of Medicine University of Pittsburgh Pittsburgh Pennsylvania USA

**Keywords:** blood‐derived materials, diabetes mellitus, glycated hemoglobin, interchangeability, processed blood material

## Abstract

**Background:**

Measurement of hemoglobin A_1C_ (HbA_1C_) is essential for the diagnosis and treatment of diabetes mellitus (DM). Processed blood materials (PBMs), such as frozen pooled blood (FPB), are commonly used for External Quality Assessment (EQA). However, their commutability may be affected by factors such as hemolysis and matrix modification. This study evaluated FPB commutability and identified the factors influencing its performance in HbA_1C_ measurements.

**Methods:**

HbA_1C_ contents in 24 clinical blood samples (CBSs) and 10 PBMs, including FPBs, single‐donor blood (SDB), and in vitro glycated blood (IGB), were analyzed using enzymatic (EN), capillary electrophoresis (CE), cation‐exchange high‐performance liquid chromatography (CE‐HPLC), turbidimetric immunoassay (TI), and boronate affinity HPLC (BA‐HPLC). EN served as the reference measurement procedure. Commutability was assessed using Deming regression and a 95% prediction interval. The hematocrit (HCT), visual appearance, plasma absorbance (PA), hemolysis index (HI), pH, and debris cells (DC) were analyzed.

**Results:**

FPB1 and SDB were commutable across all measurement procedures. FPB2 and FPB4 were commutable with CE, CE‐HPLC, and TI but non‐commutable with BA‐HPLC when HbA_1C_ was ≥ 6.23%, as data points fell outside the tolerance limits of the Deming regression lines. Non‐commutable PBMs showed higher PA and HI along with lower pH. DC affected HbA_1C_ quantification using CE‐HPLC.

**Conclusions:**

Hemolysis, as assessed by PA and HI, affects FPB commutability. These factors should be considered in EQA programs, particularly for BA‐HPLC. HCT and DC did not directly affect commutability; however, maintaining appropriate HCT levels and monitoring DC‐related chromatographic interference are crucial for ensuring FPB reliability in HbA_1C_ comparisons.

## Graphical abstract

Hemolysis increased visual appearance, plasma absorbance, and hemolysis index but did not affect the commutability of HbA1C across CE, CE‐HPLC, and TI. In contrast, BA‐HPLC results may be influenced when HbA1C exceeds 6.23% NGSP, highlighting the limitations of using frozen pooled blood (FPB) in external quality assessment. Hematocrit was not identified as a clear factor in non‐commutable blood materials; however, maintaining values similar to native blood is recommended to ensure consistency across measurement platforms.

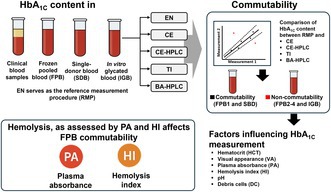

**Abbreviations**
BA‐HPLC: boronate affinity HPLC
CBS: clinical blood sample
CE: capillary electrophoresis
CE‐HPLC: ion‐exchange HPLC
EDTA: ethylenediaminetetraacetic acid
EN: enzymatic assay
FPB: frozen pooled blood materials
FWB: fresh whole blood
IFCC: International Federation of Clinical Chemistry and Laboratory Medicine
IGB: in vitro glycated blood materials
RMP: reference measurement procedures
SDB: single donor blood materials
TI: turbidimetric immunoassay

## 1 Introduction

Diabetes mellitus (DM), a major non‐communicable disease, is a growing global public health concern. According to the International Diabetes Federation, the number of adults with DM is projected to rise to 643 million by 2030 from 537 million in 2023 [1]. Thailand's Ministry of Public Health reported 3.3 million cases of DM and 16,388 DM‐related deaths in 2020 [2]. These statistics emphasize the need for accurate blood sugar measurements, as poor blood sugar control can be detrimental to various organs.

The level of hemoglobin A_1C_ (HbA_1C_), or glycated Hb, reflects the average blood sugar level over 2–3 months [3]. The American Diabetes Association recommends monitoring HbA_1C_ for DM diagnosis and management, with levels ≥ 6.5% indicating DM and between 5.7% and 6.4% indicating pre‐diabetes. Treatment aims to reduce complications by maintaining HbA_1C_ below 7% to reduce complications [4, 5, 6]. HbA_1C_ measurement does not require fasting and is therefore widely used in Thailand owing to its convenience. Various procedures have been employed for this measurement, including enzymatic assays (EN), capillary electrophoresis (CE), cation‐exchange high‐performance liquid chromatography (CE‐HPLC), turbidimetric immunoassay (TI), and boronate affinity high‐performance liquid chromatography (BA‐HPLC) [7, 8]. Standardization and quality assurance are essential to ensure accuracy. Of these tests, EN, when traceable to the International Federation of Clinical Chemistry (IFCC) Secondary Reference Measurement Procedure (SRMP), serves as the gold standard for HbA_1C_ quantification in this study.

In laboratory testing, blood materials (BM) are essential for Internal Quality Control programs and External Quality Assessment (EQA), and must comply with the International Organization for Standardization (ISO) standard 17,034: General Requirements for the Competence of Reference Material Producers, which mandates that blood materials be both homogeneous and stable before use. Additionally, materials used to compare measurement techniques must demonstrate commutability similar to that of clinical blood samples (CBSs) across different measurement techniques [9].

Fresh whole blood (FWB) is highly commutable but has limitations in terms of production volume, and processed blood materials (PBM) offer a practical alternative. In Thailand, different processing methods yield various PBMs, including frozen pooled blood (FPB), single‐donor blood (SDB), and in vitro glycated blood (IGB). Although freezing may induce lysis of red blood cells (RBC), FPB enables long‐term storage and is therefore employed in EQA programs by the Department of Medical Sciences, Ministry of Public Health [8]. WE Med Lab Center Co. Ltd. has also developed SDB and IGB materials for EQA programs [10]. PBMs must retain matrix properties similar to those of clinical blood samples (CBS); however, pooling, freezing, or the use of additives can influence commutability [11]. This study aims to assess the commutability of HbA_1C_ in PBMs and to identify factors affecting commutability to optimize PBM preparation for quality control analysis of HbA_1C_. The ultimate objective is to improve alignment between clinical laboratory standards, thereby enhancing the reliability and accuracy of HbA_1C_ testing.

## 2 Materials and Methods

### 2.1 Sample Preparation

#### 2.1.1 Clinical Blood Samples

CBSs were collected from 24 volunteers (ages 27–60) with HbA_1C_ contents ranging from 5.0% to 12.00%. FWB samples (12 mL) were collected in K3‐ethylene diamine tetraacetic acid (EDTA) tubes. The samples were stored at 2°C–8°C prior to analysis.

#### 2.1.2 Blood Materials

##### 2.1.2.1 Unprocessed Native Blood Samples

FWB materials from single‐donors were categorized into four groups based on their HbA_1C_ content: FWB1 (5.00%–5.70%), FWB2 (6.00%–7.00%), FWB3 (8.00%–9.00%), and FWB 4 (10.00%–12.00%). All samples were stored at 2°C–8°C prior to analysis.

##### 2.1.2.2 Processed Blood Material

The FPB materials were supported by the EQA program of the Department of Medical Sciences and prepared at Ramathibodi Hospital, Bangkok, Thailand, while SDB and IGB materials were prepared at the Reference Material and Medical Laboratory Innovation Research Unit, Faculty of Allied Health Sciences, Naresuan University, Phitsanulok, Thailand.

FPB materials with various HbA_1C_ contents were prepared from remanent EDTA blood and categorized into four groups: FPB1 (HbA_1C_ 5.00%–5.70%), FPB2 (HbA_1C_ 6.00%–7.00%), FPB3 (HbA_1C_ 8.00%–9.00%), and FPB4 (HbA_1C_ 10.00%–12.00%). Blood samples within each range were pooled, divided into aliquots (0.5 mL), and stored at −70°C [8].

SBD material with HbA_1C_ content ranging from 5.00% to 7.00% was obtained from individual donors, processed to adjust hematocrit (HCT) to 40% using citrate phosphate dextrose adenine (CPDA‐1) solution, and stored at 2°C–8°C [12].

IGB material was prepared by incubating donor erythrocytes with d‐glucose in phosphate‐buffered saline (PBS) containing sodium azide at 37°C, then aliquoted and stored at 2°C–8°C [12, 13].

### 2.2 Hemoglobin A_1C_ Content and Hb Typing

Analysis of the FPBs using the selected reference measurement procedure (RMP) demonstrated that EN was more similar to the target value than the other four techniques, which are not shown in this paper. Based on these findings, EN was selected as RMP. However, EN has lower specificity for measuring HbA_1C_ than CE‐HPLC and CE. Interfering substances in the sample may lead to inaccurate HbA_1C_ measurements. The λ value was used to compare replicate measurements of the 24 CBSs between the RMP and test measurement procedure.

To ensure the accuracy of the EN procedure, FPBs were sent to the International Federation of Clinical Chemistry (IFCC) in the Netherlands to determine the target value. These EQA samples, provided by the Department of Medical Sciences, were analyzed using the IFCC Secondary RMP to obtain the target value and its uncertainty. FPBs were also tested at National Glycohemoglobin Standardization Program (NGSP)‐certified laboratories in Thailand using the EN procedure to determine the mean value and uncertainty. The absolute difference ($\Delta_{m}$) between the mean EN value (*n* = 3) ($m_{1}$) and IFCC Secondary RMP target ($m_{2}$) was calculated as: $\Delta_{m}=\left|m_{1}-m_{2}\right|$. The combined uncertainty ($u_{\Delta }$), incorporating uncertainties in the laboratory testing (±2.43% NGSP) and target value (±0.09%–0.16% NGSP), was calculated using $u_{\Delta }=\sqrt{u_{m1}^{2}+u_{m2}^{2}}$ [14, 15, 16]. This combined uncertainty reflects the total measurement uncertainty of both the testing and reference laboratories. The criterion for acceptable agreement was Δ_m_ ≤ $u_{\Delta }$. In this context, if the absolute difference is less than or equal to the combined uncertainty, it indicates that the result from the EN procedure is consistent with the IFCC reference value and is considered clinically acceptable.

HbA_1C_ levels of 24 CBSs and 10 BMs were measured using five different procedures: RMP (EN) using an Alinity c (Abbott Laboratories, USA), CE using a Sebia CAPILLARYS 2 (Sebia, France), CE‐HPLC using a Tosoh G11 (Tosoh Bioscience, Japan), TI using a Cobas c513 (Roche Diagnostics, Switzerland), and BA‐HPLC using a Premier Hb 9210 (Trinity Biotech, Ireland). Each sample was analyzed in triplicate using the same reagent lot for consistency. Hb typing was performed using CE with the Sebia CAPILLARYS 2 (Sebia, France). All HbA_1C_ and Hb typing procedures were performed in NGSP‐certified laboratories [17] or those accredited under ISO 15189.

The accuracy of normal Hb typing in CBSs was determined by comparing HbA_1C_ measurements of five replicate measurements using analysis of variance (ANOVA). Post hoc analysis using the Bonferroni correction test was used if there was a statistically significant ANOVA result. The commutability study involved measuring the HbA_1C_ contents of 24 CBSs using four tandem measurement procedures: RMP‐CE, RMP‐CE‐HPLC, RMP‐TI, and RMP‐BA‐HPLC.

### 2.3 Parameter Measurements

The hematocrit (HCT), visual appearance (VA), plasma absorbance (PA), hemolysis index (HI) percentage, pH, and debris cells (DC) of commutable and non‐commutable BMs were analyzed.

HCT measurements were performed using an iFuge HCT centrifuge (Neuation). All samples were centrifuged at 400 rcf for 20 min to separate the RBCs. The absorbance (optical density) of the resulting plasma was measured using an SP‐8001 UV/Visible spectrophotometer (Metertech) with the wavelength range set between 540 and 600 nm, corresponding to the hemoglobin absorbance spectrum. The plasma pH was measured using a Humming Probe (UltraE). All measurements were conducted in triplicate to ensure accuracy, reproducibility, and control of inter‐assay variability.

The HI percentage was calculated as the ratio of the absorbance of the sample to that of the control sample at 540 nm. The control sample was prepared from hemolytic freeze‐thawed blood.

The DC percentage was determined from the integral of the peak in the chromatogram of FA (unidentifiable peak) [18] obtained using a Tosoh G11 HPLC analyzer (Tosoh Bioscience Inc., Tokyo, Japan).

### 2.4 Statistical Analysis

All statistical analyses were performed using Microsoft Excel 365 and IBM SPSS Statistics for Windows, Version 17.0 (IBM Corp., Armonk, NY, USA).

#### 2.4.1 Commutability

The RMP (EN) was traced to the IFCC Secondary RMP to validate its accuracy in measuring HbA_1C_ contents. Commutability was evaluated according to the Clinical and Laboratory Standards Institute (CLSI) guideline EP14—Evaluation of Commutability of Processed Samples, using pairwise comparisons analyzed with Deming regression [19]. BMs with values within the 95% prediction interval (PI) were considered commutable.

The lambda (λ) value in the Deming regression was calculated as the variance ratio of the measurement error between the RMP (*x*‐axis) and the test procedure (*y*‐axis) using the equation λ = ${\sigma \;}_{\in Y}^{2}$/${\sigma \;}_{\in X}^{2}$ [20, 21]. A calculated λ > 1.00 suggests that the replicate measurements of the 24 CBSs obtained using the RMP (*x*‐axis) exhibited lower error variance compared to those obtained using the test procedures, such as CE, CE‐HPLC, TI, and BA‐HPLC.

#### 2.4.2 Factors Influencing the Measurement of the HbA_1C_ Contents of PBMs

All measured parameters are presented as mean ± SD. The trends between the commutable and non‐commutable PBM groups were analyzed to identify potential factors affecting HbA_1C_ measurement.

### 2.5 Ethical Aspects

Written consent was obtained from all volunteers, and the study was approved by the Human Research Ethics Committee of Naresuan University, Thailand (COA No. 269/2023; IRB No. P1‐0093/2566).

## 3 Results

### 3.1 Commutability of HbA_1C_ Blood Materials

The Δ_m_ between the RMP (EN) and IFCC secondary RMP in FPB was within the acceptable $u_{\Delta }$ of ±2.43% NGSP (Table 1).

**TABLE 1:** Target HbA_1C_ content values in %NGSP (mmol/mol IFCC) in four FPBs and traceability of the enzymatic assay to the IFCC secondary RMP.

Blood material	HbA_1C_ content		$\Delta_{m}$ (%)	Interpretation with $u_{\Delta }$ = 2.43 (%)
	Target value mean ± uncertainty	Enzymatic assay mean ± uncertainty		
FPB1	5.21 ± 0.10 (33.4 ± 1.1)^a^	5.27 ± 2.43 (95% CI: 5.25–5.30) (34.3 ± 3.05)	0.06	Acceptable
FPB2	6.62 ± 0.09 (48.8 ± 1.0)	6.58 ± 2.43 (95% CI: 6.56–6.59) (48.3 ± 3.05)	0.04	Acceptable
FPB3	8.26 ± 0.10 (66.8 ± 1.1)	8.22 ± 2.43 (95% CI: 8.19–8.24) (66.0 ± 3.05)	0.04	Acceptable
FPB4	11.03 ± 0.16 (97.1 ± 1.8)	11.00 ± 2.43 (95% CI: 10.98–11.01) (96.7 ± 3.05)	0.03	Acceptable

Abbreviations: $\Delta_{m}$, absolute difference; $u_{\Delta }$, combined uncertainty; FPB, frozen pooled blood materials.

^a^ Indicates HbA_1C_ content expressed in mmol/mol, calculated using: HbA_1C_ (mmol/mol) = (10.93*NGSP) – 23.50.

Hemoglobin typing of 24 CBSs identified 19 cases of Hb A_2_A (normal hemoglobin, without excluding alpha‐thalassemia), 4 cases of Hb E traits, and 1 case of homozygous Hb E. Hemoglobin variants can interfere with HbA_1C_ measurement. Therefore, only the 19 CBSs with normal Hb were used to compare HbA_1C_ content across different measurement procedures. Significant differences (*p* < 0.05) were observed in the HbA_1C_ content of these 19 CBSs with normal hemoglobin across five replicate measurements (Table 2). Specifically, the HbA_1C_ content measured by the RMP (EN) differed significantly (*p* < 0.05) from those measured by CE, CE‐HPLC, TI, and BA‐HPLC. The corresponding Cohen's d effect sizes ranged from 0.08–0.13, indicating small but consistent effects.

**TABLE 2:** Analysis of HbA_1C_ content in 19 clinical blood samples using five different procedures.

Clinical blood samples	HbA_1C_ content (%NGSP)					
	RMP (EN)	CE	CE‐HPLC	TI	BA‐HPLC	*p* ^a^
Min–max	4.92–11.24	4.90–11.50	4.87–11.43	5.08–11.60	4.93–11.43	0.015
Mean	7.60	7.81^b^	7.76^b^	7.86^b^	7.81^b^	
SEM	0.45	0.45	0.46	0.46	0.47	
Median	7.15	7.47	7.33	7.44	7.33	
95% CI	6.75–8.07	6.93–8.83	6.87–8.27	6.99–8.18	6.96–8.23	
Effect sizes	NA	0.11	0.08	0.13	0.10	

*Note:* Cohen's *d* standardized mean difference (Cohen's *d* effect size) calculated using RMP (EN) as reference.

Abbreviations: BA‐HPLC, boronate affinity HPLC; CE, capillary electrophoresis; CE‐HPLC, cation‐exchange HPLC; RMP (EN), reference measurement procedure using enzymatic; SEM: standard error of the mean; TI: turbidimetric immunoassay.

^a^ Procedures were compared using replicate measurements analyzed by repeated measures analysis of variance (Repeated Measures ANOVA).

^b^ Significant differences (*p* < 0.05) from RMP (EN) in 19 CBSs were determined using the Bonferroni multiple‐comparison correction.

**FIGURE 1:**
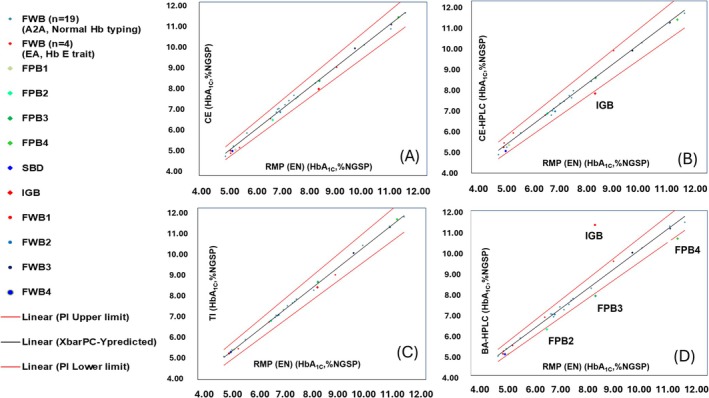
Deming regression plots comparing reference measurement procedure using enzymatic with (A) capillary electrophoresis, (B) cation‐exchange HPLC, (C) turbidimetric immunoassay, and (D) boronate affinity HPLC. FPB, Frozen pooled blood materials; FWB, Fresh whole blood materials; IGB, In vitro glycation blood materials; SBD, Single blood donor materials.

The HbA_1C_ content of 24 CBSs measured by four paired measurement procedures was analyzed using Deming regression: RMP‐CE (= 17.20), RMP‐CE‐HPLC (= 2.88), RMP‐TI (= 2.28), and RMP‐BA‐HPLC (= 16.24). A 95% PI was calculated in accordance with CLSI EP14, 4th Edition. All data are presented in Figure 1 and the commutability of the 10 BMs is summarized in Table 3. FWB1–4, FPB1, and SDB demonstrated commutability across all paired measurement techniques. FPB2 and FPB4 were commutable across RMP‐TI, RMP‐CE, and RMP‐CE‐HPLC but not across RMP‐BA‐HPLC. In contrast, IGB was non‐commutable between RMP‐CE‐HPLC and RMP‐BA‐HPLC (Table 3).

**TABLE 3:** Commutability of RMP pairwise comparison across four procedures in six PBMs.

Blood materials	HbA_1C_ content ranges from five measurements	Commutability assessment			
		CE	CE‐HPLC	TI	BA‐HPLC
SBD	5.03%–5.29%	C	C	C	C
FPB1	5.04%–5.40%	C	C	C	C
FPB2	6.23%–6.77%	C	C	C	NC
FPB3	7.85%–8.56%	C	C	C	NC
FPB4	10.64%–11.48%	C	C	C	NC
IGB	7.70%–11.30%	C	NC	C	NC

Abbreviations: BA‐HPLC, boronate affinity HPLC; C, commutable blood materials; CE, capillary electrophoresis; CE‐HPLC, cation‐exchange HPLC; FPB, frozen pooled blood materials; FWB, fresh whole blood materials; IGB, in vitro glycation blood materials; NC, non‐commutable blood materials; SBD, single blood donor materials; TI, turbidimetric immunoassay.

### 3.2 Factors Influencing the Measurement of the HbA_1C_ Contents in PBMs

**FIGURE 2:**
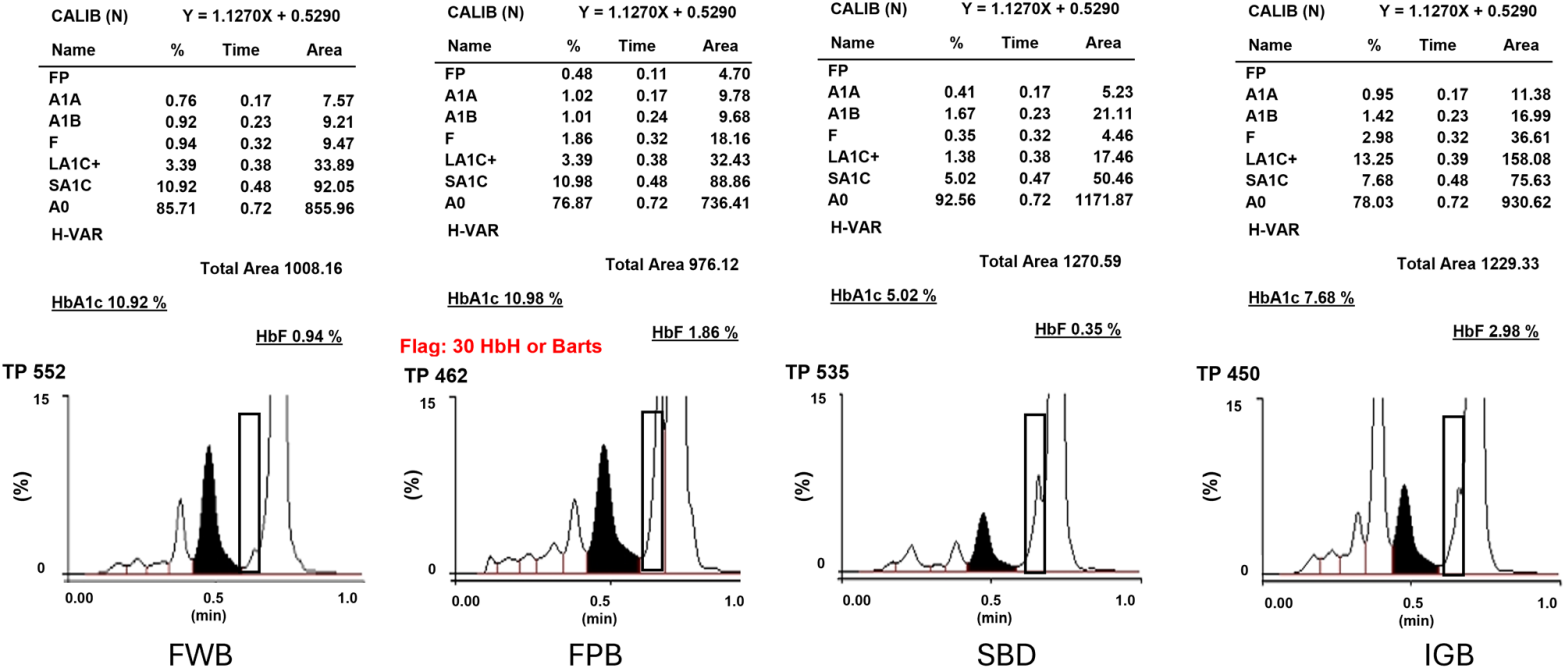
CE‐HPLC chromatogram obtained during HbA_1C_ measurement. FPB, Frozen pooled blood materials; FWB, Fresh whole blood materials; IGB, In vitro glycation blood materials; SBD, Single blood donor materials.

Table 4 compares six parameters of the commutable and non‐commutable PBMs, along with those of the unprocessed native blood samples. The PA (3.92–4.44) and HI (87%–99%) in non‐commutable PBMs were higher than those in commutable PBMs; however, the VA (4+), pH (7.00–7.68), HCT levels (0%–21%), and DC (unidentifiable peak) (0%–0.46%) were essentially identical in both commutable and non‐commutable PBMs. The PA, HI, pH, and HCT levels of the PBMs differed from those of the unprocessed native blood samples. DC (unidentifiable peak) was observed only in the FPB. Figure 2 shows the CE‐HPLC chromatographs. The A_0_ peak in the chromatograms of the PBMs differed from that of the unprocessed native blood samples.

**TABLE 4:** Measurement of parameters in ten blood materials with various HbA_1C_ by RMP.

Blood materials	Commutability	HbA_1C content_	Parameter					
			VA (Grade)	PA (Mean ± SD)	HI (%) (Mean ± SD)	pH (Mean ± SD)	HCT (%) (Mean ± SD)	DC (%) (Mean ± SD)
**Processed blood materials**								
SBD	C	5.16 ± 0.02	4+	3.89 ± 0.42	86 ± 9	7.16 ± 0.20	41 ± 0	0
FPB1	C	5.27 ± 0.02	4+	3.70 ± 0.62	82 ± 14	7.69 ± 0.10	0	0.46 ± 0.03
FPB2	NC (BA‐HPLC)	6.58 ± 0.01	4+	4.44 ± 0.05	99 ± 1	7.71 ± 0.09	0	0.46 ± 0.02
FPB3	NC (BA‐HPLC)	8.22 ± 0.02	4+	4.06 ± 0.38	90 ± 8	7.59 ± 0.08	0	0.45 ± 0.01
FPB4	NC (BA‐HPLC)	10.00 ± 0.02	4+	3.92 ± 0.54	87 ± 12	7.68 ± 0.07	0	0.49 ± 0.01
IGB	NC (CE‐HPLC, BA‐HPLC)	8.20 ± 0.03	4+	4.19 ± 0.27	93 ± 6	7.00 ± 0.08	21 ± 1	0
**Unprocessed native blood samples**								
FWB1	C	5.09 ± 0.02	2+	0.46 ± 0.11	10 ± 3	7.87 ± 0.07	39 ± 1	0
FWB2	C	6.84 ± 6.05	N	0.51 ± 0.05	14 ± 5	7.83 ± 0.05	35 ± 2	0
FWB3	C	9.47 ± 0.02	1+	0.64 ± 0.21	11 ± 1	7.95 ± 0.14	39 ± 1	0
FWB4	C	10.75 ± 0.01	N	0.67 ± 0.02	15 ± 0	8.00 ± 0.10	45 ± 0	0

Abbreviations: C, commutable blood materials; CT, hematocrit; DC, debris cell; FPB, frozen pooled blood materials; FWB, fresh whole blood materials; HI, hemolysis index; IGB, in vitro glycation blood materials; NC, non‐commutable blood materials; PA, plasma absorbance; SBD, single blood donor materials; VA, visual appearance.

## 4 Discussion

Effective DM management relies on accurate HbA_1C_ measurements, ensuring that patients receive appropriate treatment tailored to their glycemic control. Blood materials allow results to be traceable to the primary RMP and are therefore crucial in standardizing HbA_1C_ measurement across laboratories. In Thailand, commutable materials are essential for evaluating the accuracy and comparability of the diverse HbA_1C_ methodologies commonly employed in the country.

The Central Primary Reference Laboratory calibrates measurement procedures in secondary reference laboratories using pooled whole blood stored at or below −80°C [22]. Similarly, the Department of Medical Sciences employs FPB in its EQA programs to ensure consistent and reliable HbA_1C_ measurements [8]. These FPBs are traceable to the IFCC Secondary RMP, ensuring global alignment between standards. Similarly, the Royal College of Pathologists of Australasia Quality Assurance Programs (RCPAQAP) uses FWB and lyophilized blood materials in its EQA programs across Australia, New Zealand, Malaysia, Hong Kong, and Singapore; however, the RCPAQAP program is not suitable for users of the Abbott Affinion system, who are instead recommended to enroll in the FWB program [23]. Additionally, the EurA1c program incorporates FWB and lyophilized materials to assess the performance of laboratories across Asia, Europe, and Africa [17]. These BMs play a pivotal role in inter‐laboratory comparisons and standardization efforts, highlighting the necessity of commutability studies to evaluate the performance of various measurement procedures.

While previous studies have reported the influence of matrix effects and processing conditions on commutability, this study incorporates recent evidence demonstrating that freeze–thaw cycles, pooling, and storage temperature can alter sample protein conformation and increase debris, thereby compromising assay comparability [24, 25]. These mechanisms are particularly relevant to BA‐HPLC, as noted by Weykamp and Little et al., where subtle matrix alterations can disproportionately affect cis‐diol–boronate binding efficiency, leading to measurable bias.

This study assessed the commutability of PBMs, including FPBs, SBD, and IGB, using EN as the RMP. The Δ_m_ between the EN procedure and the IFCC secondary RMP was within the acceptable $u_{\Delta }$ (Δ_m_ ≤ $u_{\Delta }$), confirming traceability to the IFCC standard and reinforcing the reliability of the EN procedure for HbA_1C_ measurement (Table 1). When evaluating 19 CBSs with normal Hb typing, statistically significant differences (*p* < 0.05) were observed between EN and other procedures (CE, CE‐HPLC, TI, and BA‐HPLC) (Table 2).

The HbA_1C_ content of 24 CBSs was measured using the RMP (EN) and four measurement procedures. Deming regression of paired measurement procedures was performed based on the HbA_1C_ content of these 24 CBSs (Figure 1). The λ value was defined as the variance ratio of the measurement error between the RMP (*x*‐axis) and the respective test procedure (*y*‐axis). The calculated λ was > 1.00, suggesting that the replicate measurements of the 24 CBSs using the RMP exhibited lower error variance than those obtained using CE, CE‐HPLC, TI, and BA‐HPLC [21].

The FPB1 and SBD were commutable across all four paired measurement procedures. However, FPB2 and FPB3 were commutable when assessed using the RMP paired with TI, CE, and CE‐HPLC, but non‐commutable with BA‐HPLC. Therefore, FPBs in the EQA program, when assessed for accuracy against the RMP, should be carefully evaluated or the approach to assessing laboratory performance within the same test kit group should be adjusted. In contrast, IGB was commutable when evaluated using the RMP paired with TI, which is consistent with the findings of Duanginta et al. [13, 26]. However, IGB was non‐commutable across CE, CE‐HPLC, and BA‐HPLC (Table 3).

Matrix changes in the PBMs were analyzed based on their VA in plasma. PBMs exhibited a distinct plasma color that differed from unprocessed native blood. Elevated PA indicated increased turbidity and free Hb from lysed RBCs, while a high HI reflected extensive RBC rupture with leakage of Hb and plasma proteins. These alterations modify the sample matrix and can raise baseline noise. In BA‐HPLC, elevated PA and HI interfere with optical detection after resin‐column separation, reducing peak clarity and hindering accurate distinction of HbA_1C_ from HbA_0_. Because BA‐HPLC depends on cis‐diol–boronate binding, proteins and cellular debris from hemolysis can coat the resin or disturb column chemistry, lowering binding efficiency and potentially leading to underestimation of HbA_1C_ content. Additionally, spectrophotometric analysis, including PA and HI, revealed that plasma from non‐commutable PBMs exhibited higher light absorption than that from commutable PBMs. Similarly, HCT levels in non‐commutable PBMs were lower than those in commutable PBMs. The differences in PA, HI, and HCT of commutable and non‐commutable PBMs were attributed to hemolysis caused by the freeze‐processing method. In contrast to FWB, frozen blood is known to interfere with HbA_1C_ measurements [27]. In contrast, storing blood at −80°C does not affect HbA_1C_ measurement by CE‐HPLC [28, 29, 30]. This observation aligns with Li et al., who demonstrated that freezing‐induced hemolysis increases spectrophotometric background, and with Gislefoss et al., who described chromatographic baseline shifts and unidentifiable peaks similar to those seen in our CE‐HPLC traces [31, 32]. Improving PBM preparation remains challenging, as RBC fragility limits the extent to which hemolysis can be fully prevented during freezing. Optimizing cryopreservation protocols, adding cryoprotectants, or reducing freeze–thaw cycles may help mitigate hemolysis and reduce matrix effects in future studies. In this study, HbA_1C_ measurements were evaluated in accordance with CLSI EP4 guidelines. The FPBs were commutable across RMP‐CE, RMP‐CE‐HPLC, and RMP‐TI; however, non‐commutability with RMP‐BA‐HPLC was observed when the HbA_1C_ content exceeded 6.23% NGSP. This observation was attributed to sample turbidity, which may have interfered with light absorption during HbA_1C_ measurement, potentially affecting the comparability between the RMP and BA‐HPLC.

The impact of HCT on HbA_1C_ measurement remains unclear. In this study, FPB1, with an HCT of 0%, was commutable across all procedures. However, extremely low HCT levels may pose challenges for certain measurement methods and require caution in interpretation. While HCT does not directly affect the commutability of PBMs in this study, it can influence the accuracy of HbA_1C_ measurements. When HCT is reduced due to hemolysis, the proportion of RBCs decreases. This may cause alterations in the behavior of measurement assays that rely on the precise volume of RBCs. Such changes may result in the misestimation of HbA_1C_ content, as the reagent volume may no longer align with the intended calibration for accurate testing.

BA‐HPLC demonstrated pH‐dependent effects, with a low pH (7.00) in the IGB. The commutable group exhibited higher pH levels (7.16–7.69) than the non‐commutable group (pH 7.00). pH is known to influence the binding of cis‐diol groups and glycated Hb in BA‐HPLC, with optimal interactions occurring in wash buffer at pH 7.75–8.25 (optimum pH 8.0) [33]. Although BA‐HPLC is less sensitive to pH variations than CE‐HPLC in the separation of HbA_1C_ and HbA_0_, PBMs with a pH lower than that of unprocessed blood may influence HbA_1C_ binding in BA‐HPLC.

DC analysis of FPB revealed FA peak and unidentifiable peaks due to sample degradation during prolonged blood storage. An increased P0 peak in the chromatograms of PBM also affected %HbA_1C_ calculation using CE‐HPLC. Laboratory guidelines recommend investigating unidentifiable peaks (FA and P0) before reporting results [18]. A previous study observed similar peaks before P_0_ in chromatograms of blood stored at 37°C; however, such peaks were not observed in the chromatograms of blood stored at −84°C for 18 months [34], suggesting these peaks arise from storage conditions rather than sample preparation. Nevertheless, the FPB remained commutable between RMP and CE‐HPLC. Prolonged storage leads to Hb degradation, which interferes with CE‐HPLC measurements; however, the cis‐diol group on glucose‐modified Hb remains intact, enabling binding to the BA column resin without interference [31].

To improve the commutability and reliability of PBMs, future preparation criteria should include optimizing storage conditions, maintaining HCT levels, stabilizing pH, and minimizing chromatographic interference. FPBs should be stored at −70 to −80°C to prevent hemolysis and minimize degradation before pooling. HCT levels in SDB and IGB should be maintained between 36% and 54%, similar to those in FWB [35]. A 0.1 M sodium hydroxide solution should be used to maintain a pH between 7.83 and 8.00 for BA‐HPLC.

We acknowledge that the sample size of this study was limited, which may affect the generalizability of the conclusions. Due to inherent challenges in producing PBMs—including stability, availability, and cost‐ it was not feasible to include a larger number of samples. Therefore, the results should be interpreted as indicative trends rather than definitive conclusions. Nonetheless, despite the relatively small dataset, this study identified that increased PA and HI, resulting from hemolysis, are key factors affecting commutability. By contextualizing these findings alongside recent literature on matrix effects and assay‐specific vulnerabilities [24, 25, 31, 32], the present work not only corroborates earlier observations but also highlights unique patterns in BA‐HPLC susceptibility observed under the conditions of this study. Future advancements will require the generation of larger quantities of PBMs, incorporating comparisons across blood materials with varying PA, HI, and pH levels, and applying robust statistical analyses to ensure accuracy and reliability.

## 5 Conclusion

Homolysis increased visual appearance (VA), plasma absorbance (PA), and hemolysis index (HI) levels; however, HbA_1C_ content was unaffected, enabling commutability across capillary electrophoresis (CE), cation‐exchange high‐performance liquid chromatography (CE‐HPLC), and turbidimetric immunoassay (TI). In contrast, these factors may affect HbA_1C_ measurement by boronate affinity high‐performance liquid chromatography (BA‐HPLC) at HbA_1C_ contents greater than 6.23% NGSP. Therefore, specific limitations should be acknowledged for HbA_1C_ measurement using BA‐HPLC when frozen pooled blood (FPBs) materials are used as external quality assessment (EQA) samples. Haematocrit (HCT) could not be definitively identified as a factor affecting HbA_1C_ measurement in non‐commutable blood; however, maintaining HCT levels similar to those of unprocessed native blood is recommended to ensure compatibility across all analytical platforms. Additionally, the presence of unidentifiable peaks, likely resulting from increased debris cell (DC) owing to prolonged sample storage of FPBs, may contribute to measurement inaccuracies and should, therefore, be carefully considered.

## Author Contributions

Kanokwan Ngueanchanthong: data curation (equal), formal analysis (lead), writing – original draft (lead), writing – review and editing (lead). Napaporn Apiratmateekul: methodology (lead), writing – review and editing (lead). Supaporn Suparak: methodology (lead), writing – review and editing (lead). Nam K. Tran: formal analysis (lead), methodology (lead), writing – review and editing (lead). Wanvisa Treebuphachatsakul: data curation (equal), formal analysis (lead), funding acquisition (equal), investigation (lead), methodology (lead), resources (equal), validation (equal), writing – original draft (lead), writing – review and editing (lead).

## Disclosure

Portions of this data were previously presented in poster format at the APFCB 2024 conference in Australia, with publication support provided by the event organizers.

## Conflicts of Interest

The authors declare no conflicts of interest.

## Supporting information


**Data S1:** jcla70125‐sup‐0001‐Supinfo01.pdf.

## Data Availability

The data sets generated and/or analysed in the current study are not publicly available for ethics reasons, but can be requested from the corresponding author upon reasonable request.
