# Supplementary material for: Investigating the Factors Affecting the Commutability of Hemoglobin A1C Frozen Pooled Blood Materials
Source: J Clin Lab Anal. 2025 Oct 31;39(23):e70125. doi: 10.1002/jcla.70125 (PMC12699204; doi:10.1002/jcla.70125)
Supplement: Supplementary file 1 — Data S1: jcla70125‐sup‐0001‐Supinfo01.pdf. [file JCLA-39-e70125-s001.pdf]

Table S1 Commutability of RMP pairwise comparison across four procedures in six PBMs

Blood materials	HbA1C content				
	**EN**	**CE**	**CE-HPLC**	**TI**	**BA-HPLC**
SBD	5.16 ± 0.02	5.13 ± 0.06	5.03 ± 0.06	5.29 ± 0.02	5.03 ± 0.08
FPB1	5.27 ± 0.02	5.20 ± 0.10	5.30 ± 0.10	5.40 ± 0.02	5.04 ± 0.02
FPB2	6.58 ± 0.01	6.57 ± 0.15	6.77 ± 0.06	6.75 ± 0.02	6.23 ± 0.04
FPB3	8.22 ± 0.02	8.37 ± 0.06	8.43 ± 0.06	8.56 ± 0.04	7.85 ± 0.07
FPB4	11.00 ± 0.02	11.30 ± 0.17	11.13 ± 0.12	11.48 ± 0.09	10.64 ± 0.19
IGB	8.20 ± 0.03	8.00 ± 0.10	7.70 ± 0.00	8.31 ± 0.04	11.30 ± 0.13

Abbreviations: BA-HPLC: boronate affinity HPLC; C: commutable blood materials; CE: capillary electrophoresis; CE-HPLC: cation-exchange HPLC; EN: enzymatic assay; FPB: frozen pooled blood materials; FWB: fresh whole blood materials; IGB: in vitro glycation blood materials; NC: non-commutable blood materials; SBD: single blood donor materials; TI: turbidimetric immunoassay
